# Gravidity is not associated with telomere length in a biracial cohort of middle-aged women: The Coronary Artery Risk Development in Young Adults (CARDIA) study

**DOI:** 10.1371/journal.pone.0186495

**Published:** 2017-10-19

**Authors:** Abbi D. Lane-Cordova, Eli Puterman, Erica P. Gunderson, Cheeling Chan, Lifang Hou, Mercedes Carnethon

**Affiliations:** 1 Department of Preventive Medicine, Feinberg School of Medicine, Northwestern University, Chicago, Illinois, United States of America; 2 School of Kinesiology, University of British Columbia, Vancouver, British Columbia, Canada; 3 Division of Research, Kaiser Permanente Northern California, Oakland, California, United States of America; Univesity of Iowa, UNITED STATES

## Abstract

**Objective:**

Having experienced 2–3 births is associated with reduced mortality versus women with <2 or ≥4 births. The effect of 2–3 births on lifespan may be associated with delayed cellular aging. We hypothesized telomere length, a marker of cellular aging, would be longer in women who had 2–3 pregnancies.

**Methods:**

Leukocyte telomere length was measured using quantitative real-time polymerase chain reaction in 620 women in CARDIA at the year 15 and 20 exams, expressed as the ratio of telomere repeat copy number to single-copy gene copy number (*T*/*S*). Number of pregnancies at the time of telomere length measurement was obtained (mean age = 41±0.1 years, average gravidity = 2.64±0.1 pregnancies). Participants were divided into 4 groups by number of pregnancies: 0, 1, 2–3, and ≥4, to test for differences in telomere length by gravidity group.

**Results:**

The mean and SD for telomere length was 0.98 ± 0.20 *T/S* in the whole cohort. There were no differences in mean telomere length between groups; 0.98±0.02 *T/S* in women with 0 pregnancies, 1.01±0.02 *T/S* in women with 1 pregnancy, 0.97±0.01 *T/S* in women with 2–3 pregnancies, and 0.99±0.02 *T/S* in women with ≥4 pregnancies (p = 0.51). We defined high-risk (shorter) telomere length as ≤25^th^ percentile, and low-risk (longer) telomere length as ≥75 percentile. There were no differences in the prevalence of high-risk or low-risk telomere length between gravidity groups.

**Conclusions:**

Gravidity was not associated with telomere length in early middle age; the protective association of 2–3 births may act through other mechanisms.

## Introduction

Pregnancy has lasting effects on vascular function, such as reducing arterial stiffness and mean arterial pressure [1]. Parity, the number of times a woman has experienced a pregnancy lasting >20 weeks, is also related to cardiovascular disease mortality in a U-shaped pattern. Women who have had 2 or 3 births have the lowest cardiovascular mortality rate compared with women with less than 2 or more than 4 births [2]. Furthermore, women who have had 2–3 births have less evidence of subclinical atherosclerosis (reduced carotid intima-media thickness; cIMT, coronary artery calcium; CAC, and increased carotid distensibility) compared with women who have had less than 2 or 4 or more births years later [3–5]. Taken together, these data suggest that experiencing 2–3 pregnancies may reduce cardiovascular risk during aging in women.

Telomeres are comprised of nucleoproteins that form a cap at the end of chromosomes, and shorter telomere length indicates increased cell division [6]. Telomere length can be quantified as a measure of biological cellular aging, and shorter telomere length has been associated with mortality in women [7, 8]. Shorter telomere length has also been linked to increased subclinical atherosclerotic burden (CAC and arterial stiffness) [9, 10], but telomere length attrition is associated with vascular aging independent of traditional cardiovascular risk factors [11]. This is important because both traditional cardiovascular risk factors and subclinical atherosclerosis are thought to damage the cardiovascular system and lead to overt disease. The protective effect of 2–3 pregnancies on overall and cardiovascular mortality may be due to reduced cellular aging, evident by longer telomere length, in women who have had 2–3 pregnancies.

The purpose of this study was to determine whether there is an association between number of pregnancies (gravidity) and mid-life telomere length in women. We hypothesized that women who have had 2–3 pregnancies would have longer telomeres compared with women who have had less than 2 or ≥ 4 pregnancies. We further hypothesized that the strength of the relationship between number of pregnancies and telomere length would be reduced after adjustment for other cardiovascular risk factors as telomere length has been associated with CVD [9, 10]. Short telomere length (≤25^th^ percentile) has been associated with multiple biological and behavioral risk factors for atherosclerotic cardiovascular disease [12–14]; we also hypothesized that women who have had 2–3 pregnancies would be less likely to have short telomere lengths, defined as ≤ 25^th^ percentile, compared to women with <2 or ≥4 pregnancies. Telomere length and telomerase activity can be acutely altered during pregnancy in certain pregnancy-related conditions [15, 16], so we also tested for differences in telomere length in women with and without gestational diabetes and preterm birth.

## Methods

The CARDIA Study is a longitudinal, observational, population-based, multi-center, study investigating determinants of coronary heart disease risk factors in African-American and Caucasian men and women. In 1985–1986, 5115 subjects (53% women, 52% African-American) aged 18 to 30 years were recruited from 4 urban areas in the United States: Birmingham, Alabama; Chicago, Illinois; Minneapolis, Minnesota; and Oakland, California. Retention rates at follow-up exams 15 and 20 years later (2000–2001 and 2005–2006) were 74%, and 72% of the surviving cohort, respectively. Institutional review boards at each study center approved the study and written, informed consent was obtained from participants.

### Sample selection

Telomere length was measured in 620 women from an ancillary study in which participants were selected if they had stored DNA from whole blood available at Y15, Y20 and Y25 and also had coronary artery calcification data on the same examinations. Participants in the ancillary study from which we drew our sample were selected so that there would be a similar number of participants by race, education, sex, and study center. For those 1000 participants (620 women), telomere length was determined. In this study, we split the women in the telomere cohort into 2 groups according to age at baseline so that we could construct a sample with the same mean age because age is a main determinant of telomere length. We included the telomere length measurement at Y15 in the women who were older at baseline (24–30 yrs) and telomere length measurement at Y20 in the women who were younger at baseline (18–23 yrs) so that each woman was between 38 and 45 yrs old at the time of the measurement. Women included in the sample were younger, had higher triglycerides, more likely to be on cholesterol-lowering medication, and had a lesser percentage of preterm birth compared to women not in the sample, Table 1.

**Table 1:** Participant characteristics compared women not in sample.

	Telomere Sample N = 620	Women Not in Sample N = 1326
Age (yrs)*	41.5 ± 0.1	42.1 ± 0.1
African-American (n, %)	290, 47	330, 25
Education (yrs)	15 ± 0.1	15 ± 0.1
Smoking (pack-yrs)	0.60 ± 0.06	0.56 ± 0.04
Alcohol (ml/day)	6.8 ± 0.5	7.5 ± 0.6
BMI (kg/m^2^)	29.2 ± 0.3	29.5 ± 0.3
Hypertension Meds (n, %)	69, 11	163, 12
SBP (mmHg)	111 ± 1	112 ± 1
DBP (mmHg)	72 ± 1	72 ± 1
Cholesterol Meds (n, %)*	23, 4	27, 2
Cholesterol (mg/dL)	183 ± 1	182 ± 1
LDL-C (mg/dL)	108 ± 1	107 ± 1
HDL-C (mg/dL)	55 ± 1	57 ± 1
Triglycerides (mg/dL)*	94 ± 2	91 ± 2
Diabetes (n, %)	21, 3	67, 5
GDM (n, %)	33, 5	69, 5
PTB (n, %)*	77, 12	219, 17

Sample characteristics compared to women not the telomere sample. Education: years of formal schooling, Smoking: pack-yrs, a cumulative smoking score; 1 pack-yr = 7000 cigarettes, Hypertension Meds: percentage of women taking medication for high blood pressure, SBP: systolic blood pressure, DBP: diastolic blood pressure, Cholesterol Meds: percentage of women taking medication for high cholesterol, LDL-C: low-density lipoprotein cholesterol, HDL-C: high-density lipoprotein cholesterol, Diabetes diagnosis of diabetes mellitus, GDM: diagnosed gestational diabetes, PTB: preterm birth, any birth occurring before 37 weeks gestation.

*indicates a difference between groups. Data are mean ± SE unless otherwise indicated.

### Gravidity

Women reported the number of pregnancies and the length of gestation at each exam. Participants also reported current pregnancy status and the number of pregnancies at each exam. Total number of pregnancies (gravidity) at year 15 or 20 (the year at which telomere length was measured) was used for analysis.

### Blood draw and DNA extraction

Blood samples were obtained with venipuncture at exams in year 15 (for CARDIA participants aged 24–30 yrs at baseline) and year 20 (for CARDIA participants aged 18–23 at baseline) following an overnight fast (>8 h) with EDTA-containing tubes. Participants were asked not to perform heavy exercise or smoke in the two hours preceding the visit. Whole blood was stored at -70°C and shipped on dry ice to Dr. Fornage’s Genetics lab at University of Texas Health Science Center (Houston, TX) for DNA preparation. Peripheral blood mononuclear cells (PBMCs) were obtained from whole samples using Ficoll-Paque PLUS (Amersham) according to manufacturer's instructions, cryopreserved, and stored in liquid nitrogen until assays were performed. DNA was prepared using Gentra Puregene Cell kit (QIAGEN, Valencia, CA, USA) according to manufacturer’s instructions.

### Telomere length

The CARDIA study shipped DNA samples in 96-well plates to the lab of Dr. Blackburn at University of California-San Francisco, where these plates were stored at -80°C. Five ul of the DNA was diluted with 10 ul of sterile water so that the concentration was 33.3 ng/ul. Diluted samples were stored at -80°C in 96-well plates until assayed. Then, they were transferred to 384-well plates after being thawed on ice. The telomere length measurement assay was adapted from the original method, detailed elsewhere [17]. Telomere length was measured using DNA with a quantitative polymerase chain reaction (qPCR) assay. The assay determines the ratio of telomere repeat copy number to single-copy gene copy number (*T*/*S*). *T/S* was measured in our samples and compared to a reference sample of DNA. A higher *T*/*S* ratio indicates longer telomere length. *T/S* were converted to base pairs using the equation: 3274+2413x *T/S* [17]. The coefficient of variation for the assay was 3.5%.

### Co-variable measurements

Body mass index was calculated by dividing the weight in kilograms by height in meters squared. Blood pressure was measured in the right arm with a sphygmomanometer following a standardized rest period. Education, smoking, diagnosis of diabetes, gestational diabetes, use of hypertension and cholesterol-lowering medications were determined by self-report. Preterm birth (PTB) was defined as any birth occurring at <37 weeks of gestational age, according to self-report. Alcohol consumption (ml/day) was quantified as the number of drinks per week multiplied by ethanol concentration and included because alcohol has been shown to affect telomere length. Total cholesterol, LDL (calculated with the Friedewald equation), HDL cholesterol and triglycerides were measured in plasma following a fast. For a more detailed description of data collection, please visit the CARDIA website: http://www.cardia.dopm.uab.edu/

### Statistical analyses

Participants were divided into 4 groups by number of pregnancies: 0, 1, 2–3, and ≥ 4 pregnancies. Group differences in participant characteristics were assessed with ANOVA, chi-square, and Mann-Whitney U tests for skewed data, as appropriate. We tested for differences in log-transformed mean telomere length between groups using ANOVA. We used multiple linear regression to test for associations between gravidity and telomere length (adjusted for age and race). Because the groups had different years of education and proportions of women with gestational diabetes and preterm births and all these factors have been associated with telomere length in earlier investigations [15, 16, 18], we performed an ANCOVA to determine whether years of education, preterm birth, or gestational diabetes affected the association of telomere length and gravidity group. A chi-square test was performed to determine whether there was a difference in prevalence of high-risk, short telomere length, defined as the lowest quartile of telomere length (≤ 0.844) between groups. We also evaluated whether there was a difference in prevalence of low-risk, long telomere length, defined as the highest quartile, ≥ 1.098, between groups. We determined whether there was a difference in likelihood of having a high-risk or low-risk telomere length by gravidity group using logistic regression. We repeated all analyses stratified by race, as race has been shown to influence telomere length in women [18]. We also tested whether women with gestational diabetes or preterm birth had shorter telomeres than women without these complications in our sample because telomerase activity has been shown to be acutely affected by complications of pregnancy [15]. Next, we conducted two sensitivity analyses. First, we tested for the effect of parity (versus gravidity) by conducting an analysis in the same women after reclassifying them into the same four categories after subtracting any pregnancies lasting < 20 weeks from their total number of pregnancies. Second, we repeated our analyses after omitting participants with telomere length that was greater than 3SD from the mean. Data are presented as means and standard errors. A p-value less than 0.05 was considered significant. Analyses were conducted using STATA version 14.0 (STATA Corporation, College Station, TX).

## Results

When we compared women by gravidity group, we observed similar rates of hypertension and diabetes. Blood pressure, blood chemistries, and smoking histories were also similar among groups. The women in the higher gravidity groups tended to have completed fewer years of education, had a greater percentage of percent of African-American women, and greater percentage of women with gestational diabetes, Table 2.

**Table 2:** Participant characteristics at time of telomere length measurement by gravidity group.

	0 Pregnancy	1 Pregnancy	2–3 Pregnancies	≥4 Pregnancies
N	101	83	264	172
Age (yrs)	41 ± 0.3	42 ± 0.3	41 ± 0.2	42 ± 0.2
African-American (n, %)†	33, 33	36, 40	119, 45	102, 59
Education (yrs)†	16 ± 0.2	15 ± 0.2	15 ± 0.1	14 ± 0.2
Smoking (pack-yrs)	0.37 ± 0.1	0.69 ± 0.2	0.57 ± 0.1	0.74 ± 0.1
Alcohol (ml/day)	7.5 ± 1.4	6.8 ± 1.2	6.8 ± 0.7	6.7 ± 1.0
BMI (kg/m^2^)	29.1 ± 0.7	28.7 ± 0.6	29.3 ± 0.4	29.6 ± 0.6
Hypertension Meds (n, %)	7, 7	10, 12	30, 11	21, 12
SBP (mmHg)	110 ± 1	113 ± 2	110 ± 1	112 ± 1
DBP (mmHg)	71 ± 2	73 ± 2	71 ± 1	72 ± 1
Cholesterol Meds (n, %)	5, 5	2, 2	9, 3	7, 4
Cholesterol (mg/dL)	185 ± 3	190 ± 4	182 ± 2	181 ± 2
LDL-C (mg/dL)	110 ± 3	113 ± 3	107 ± 2	108 ± 2
HDL-C (mg/dL)	56 ± 1	57 ± 2	55 ± 1	55 ± 1
Triglycerides	92 ± 4	98 ± 6	93 ± 4	95 ± 6
Diabetes (n, %)	3, 3	0, 0	12, 5	6, 3
GDM (n, %)†	0, 0	2, 2	8, 3	6, 3
PTB (n, %)†	0, 0	4, 5	34, 13	39, 23
Telomere length ≤25% (n,%)	29, 29	19, 23	73, 28	35, 20
Telomere length ≥75% (n,%)	20, 20	25, 30	63, 24	47, 27

Subject characteristics by gravidity group. Education: years of formal schooling, Smoking: pack-yrs, cumulative smoking score, 1 pack-yr = 7000 cigarettes, Hypertension Meds: percentage of women taking medication for high blood pressure, SBP: systolic blood pressure, DBP: diastolic blood pressure, Cholesterol Meds: percentage of women taking medication for high cholesterol, LDL-C: low-density lipoprotein cholesterol, HDL-C: high-density lipoprotein cholesterol, Diabetes diagnosis of diabetes mellitus, GDM: diagnosed gestational diabetes, PTB: preterm birth, any birth occurring before 37 weeks gestation, Telomere length ≤ 25%: high-risk, shortened telomere length ≤ 25^th^ percentile for the cohort, Telomere length ≥ 75%: low-risk, longer telomere length ≥ 75^th^ percentile for the cohort.

^†^Indicates a difference between groups. Data are mean ± SE unless otherwise indicated.

**Fig 1:**
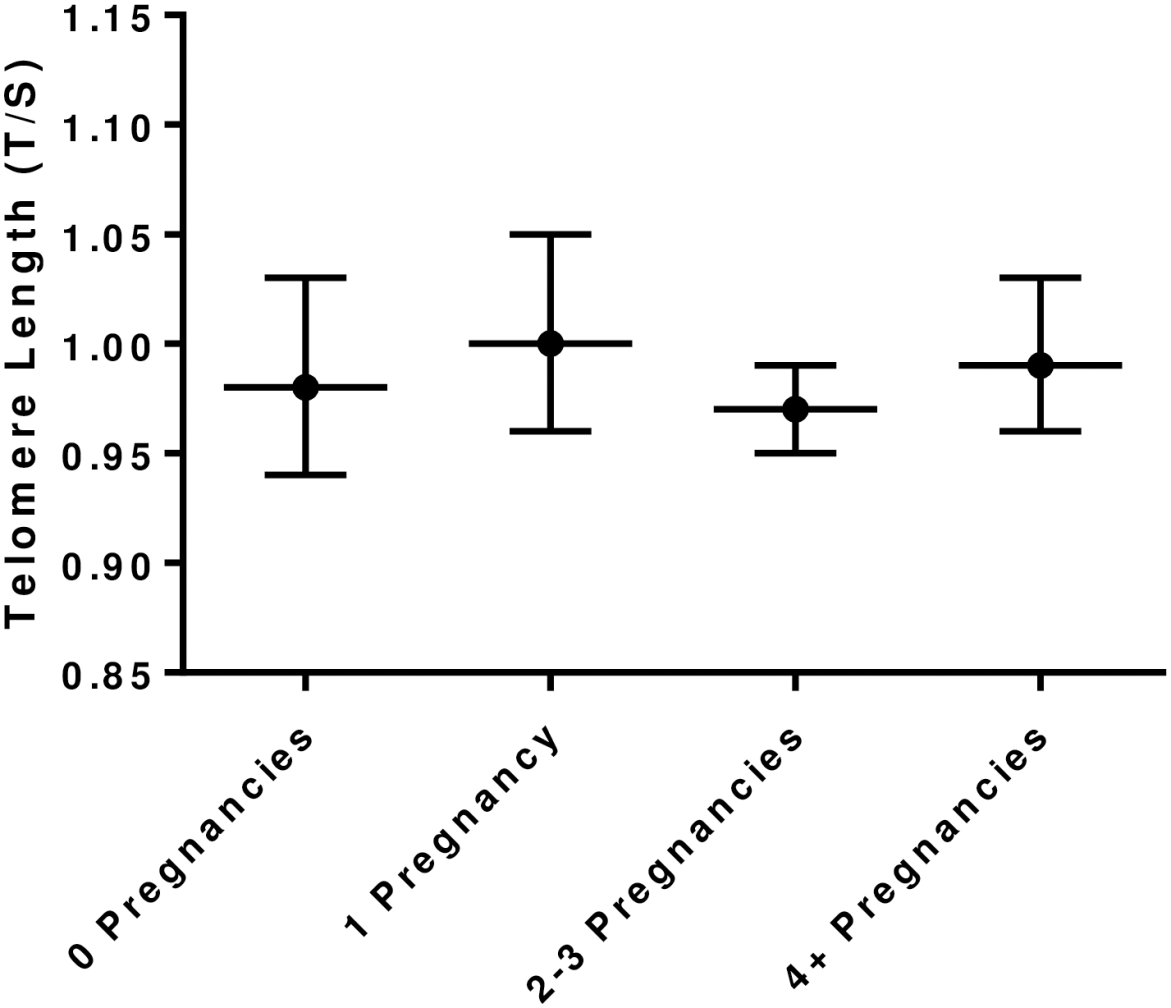
Telomere length (mean and 95% CI) by gravidity group. There were no differences in unadjusted telomere length between gravidity groups.

The overall mean and SD for telomere length was 0.98 ± 0.20 *T/S*. There was no difference in unadjusted mean telomere length between the four gravidity groups, p = 0.51, Fig 1.

Telomere length was the same in the women who had had 2 or 3 pregnancies versus all the other women, mean telomere length = 0.97 ± 0.01 *T/S* in women with 2 or 3 pregnancies and mean telomere length = 0.99 ± 0.01 *T/S* in all other women, p = 0.34. There was no difference in telomere length between women who were nulliparous versus those who had had at least one pregnancy: mean telomere length = 0.98 ± 0.01 *T/S* in nulliparous women and mean telomere length = 0.98 ± 0.02 *T/S* in women who had had a pregnancy, p = 0.42.

**Fig 2:**
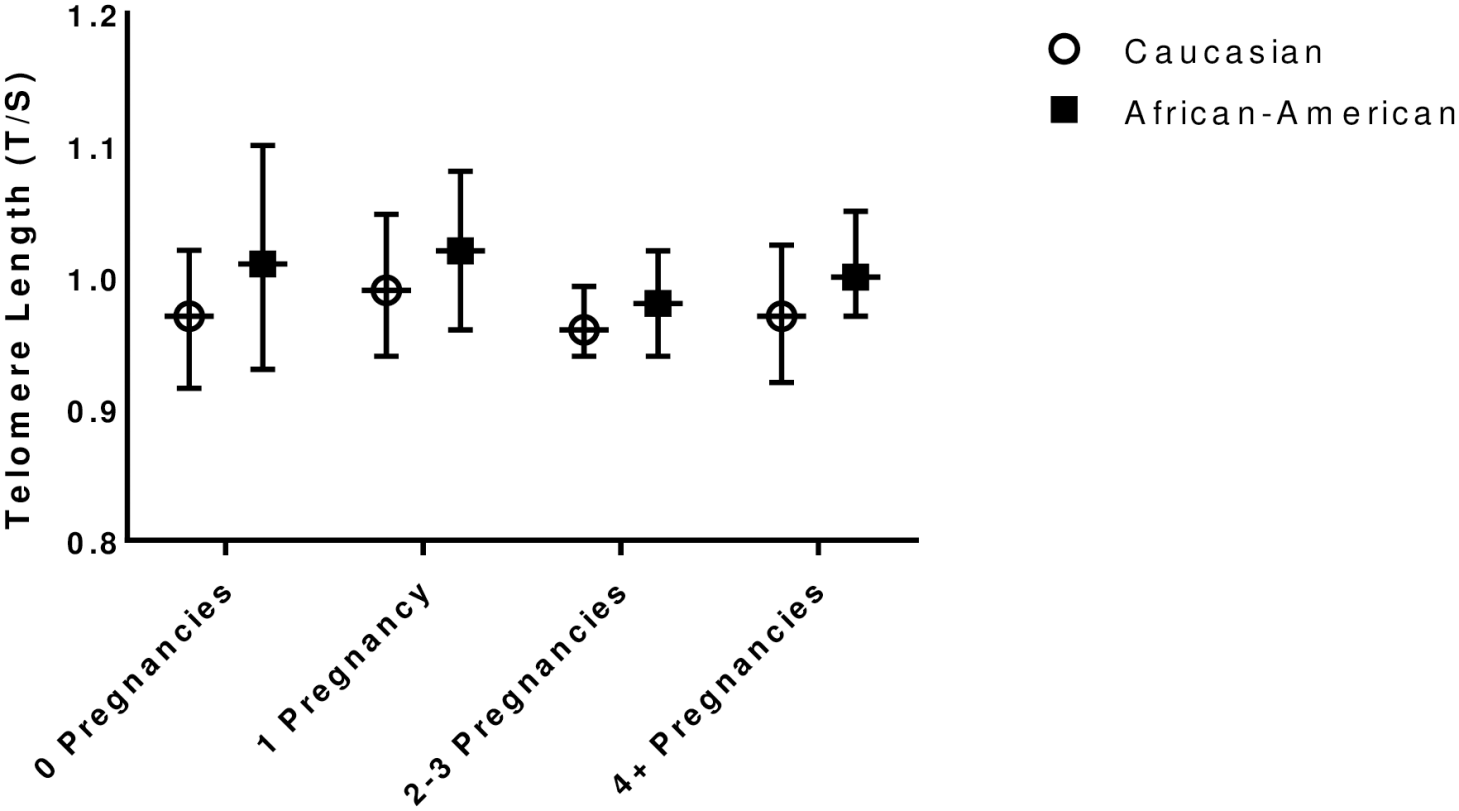
Telomere length (mean and 95% CI) by gravidity group in Caucasian and African-American women. There were no differences in unadjusted telomere length between gravidity groups in either race.

Mean telomere length did not differ by gravidity group in either African-American or Caucasian women, Fig 2, p = 0.55 for the difference between gravidity groups in African-American women, and p = 0.85 for the difference in telomere length between gravidity groups in Caucasian women. However, the effect of race on mean telomere length approached significance, 0.97 ± 0.01 in Caucasian and 1.00 ± 0.01 *T/S* in African-American women, p = 0.07.

Furthermore, there was no association between gravidity group (β = 0.002 ± 0.01, p = 0.72), years of education (β = 0.0009 ± 0.003, p = 0.80), preterm birth (β = 0.01 ± 0.03, p = 0.63), or gestational diabetes status (β = -0.020 ± 0.04, p = 0.58), with telomere length.

One hundred fifty-six women had high-risk telomere length, defined as telomere length ≤ 0.8445 *T/S*, the cut-off for the 25^th^ percentile. There was no difference in prevalence of high-risk telomere length between gravidity groups, p = 0.27, Table 2. One-hundred fifty-five women had a low-risk telomere length ≥ 1.099 *T/S*, the cut-off for the 75^th^ percentile for the cohort, with no difference in prevalence of low-risk telomere length among gravidity groups, p = 0.35, Table 2.

In our first sensitivity analysis, there was no association of gravidity group with the likelihood of having high-risk (β = -0.099 ± 0.07, p = 0.14) or low-risk (β = 0.046 ± 0.07, p = 0.49) telomere length. Having had 0, 1, or ≥ 4 pregnancies was not associated with increased or decreased odds of having high-risk or low-risk telomere length after adjustment for age and race, Table A in S1 File and Table B in S1 File.

Next, we reclassified all women into parity groups (0, 1, 2–3, ≥ 4) after omitting any pregnancy that lasted less than 20 weeks from each woman’s total number of pregnancies to test for the effects of parity/births on telomere length. There were 174 total pregnancies lasting less than 20 weeks: 13 women who had reported 1 pregnancy, 69 women who had reported 2–3 pregnancies, and 92 women who had reported 4 or more pregnancies. There was also no difference in telomere length among these parity groups: 0.99 ± 0.02 in women with 0 births; 1.0 ± 0.02 in women with 1 birth; 0.98 ± 0.01 in women with 2–3 births; and 0.98 ± 0.02 *T/S* in women with 4 or more births; p = 0.43 for the difference between groups. There was no difference in mean telomere length by parity when we stratified by race, p = 0.27 in African-American and p = 0.37 in Caucasian women.

When we tested for differences in mean telomere length between gravidity groups after omitting subjects with telomere length >3 SD from the mean, (n = 596) the results were the same. In these women, telomere length was similar in all groups: 0.94 ± 0.02 in women with 0 pregnancies; 0.99 ± 0.02 in women with 1 pregnancy; 0.96 ± 0.01 in women with 2–3 pregnancies; and 0.97 ± 0.01 *T/S* in women with 4 or more pregnancies; p = 0.23 for the difference between groups.

## Discussion

Our study showed that mean telomere length did not vary between women with different numbers of pregnancies or differences in parity in our middle-aged cohort. There was no difference in the prevalence of high or low-risk telomere length between gravidity groups and no difference in cardiovascular risk factor profiles between gravidity groups. These results persisted when we analyzed African-American and Caucasian women separately, indicating that there is no effect of gravidity on mid-life telomere length in either Caucasian or African-American women. Our study confirmed racial differences in telomere length between Caucasian and African-American women [18, 19], but we report for the first time that differences in gravidity or parity do not explain the race difference in telomere length in mid-life.

Notably, some earlier studies in telomere length were conducted in postmenopausal women, and the mean ages in these earlier investigations were in the early 60s and late 50’s. The average age in our study was 42 years. This is likely an important distinction as rate of telomere length attrition is rapid from birth to age 18 and then speeds up again after age 45, in late-middle life [11]. Measuring telomere length later in life (past age 45) may reveal differences in telomere length between gravidity groups that were not apparent at a younger age. Measurement later in life may also capture differences in clinical characteristics between groups. In our study, there also were no difference in traditional cardiovascular risk factors between the gravidity groups (Table 2).

Although the prior studies included age in their models, the difference in age at which telomere length was measured between our populations is important. Due to the non-linear pattern of telomere attrition, statistical adjustment for age may be inadequate. Indeed, the authors of previous related studies all reported that shorter telomere length was associated with age [18, 19]. Therefore, a strength of our study is the large sample size that allowed us to group subjects to ensure all participants minimize age differences at the time of telomere length measurement.

Although telomere length has been independently associated with cardiovascular disease, it has also been linked to social factors that influence cardiovascular outcomes and quality of life. Neighborhood social environment [20], neighborhood quality (a metric including crime, noise, and fear) [21], race discrimination [22], and education level [23] have all been associated with leukocyte telomere length. Telomere length is also associated with recent (< 6 yr ago) stressful life events [24]. In another study conducted in young-middle aged women (25–55 years), telomere length was associated with altered left ventricular filling dynamics but not with systolic function or left ventricular mass [25]. In our study, any group differences in education, prevalence of gestational diabetes, or preterm birth also did not influence our findings, despite the acute effects of pregnancy complications on telomerase activity [15].

Barha and colleagues hypothesized that social structure could moderate the effect of gravidity on telomere length in women [26]. In their study of 75 race/ethnically homogenous women with similar lifestyle habits, they found that the association between higher gravidity and longer telomere length approached significance, contrary to their a priori hypothesis that more child-bearing would be associated with greater cellular aging. They postulated that women who had more children may have developed a greater social support networks that buffered any detrimental effects of reproduction on telomere length [26]. Taken together, the results of these physiological and psychological studies suggest the influence of social factors and traditional cardiovascular risk factors are likely more important than gravidity alone in determining telomere length in early middle age in women.

A limitation of the study is the measurement of telomere length at a single time point in early middle age. Serial measurements would have allowed us to determine the rate of telomere shortening. Measuring telomere length later in life may allow the impact of life events from ~18–45 years (including gravidity) to affect telomere length more substantially and yield more separation in telomere length between groups. Not all women in CARDIA completed child-bearing by the Y20 exam, but there were only twenty-six new births reported in all of CARDIA at the Y25 exam, versus 132–546 new births reported at each of the Y0-Y20 exams. Our sample included only 620 of all CARDIA women, and so the subset of women with an additional birth in our study is quite small and likely minimally impacted our findings. Further, an earlier study found that maternal age at first birth was not independently associated with telomere length [26]. We tested for differences in telomere length between women who had had preterm birth and gestational diabetes compared to those who did not and found no difference, but this result should be confirmed in a larger cohort as we were underpowered to detect modest differences.

### Conclusions

Gravidity is not associated with telomere length in our biracial cohort of women in early middle age. The protective effects of 2–3 pregnancies on overall and cardiovascular mortality do not appear to be mediated by reduced cellular aging in mid-life. Further investigations should examine the effects of gravidity on mean telomere length later in life, once the rate of telomere attrition has increased, or the effect of gravidity on the rate of telomere shortening from early adulthood to middle or older age.

## Supporting information

S1 File Low and high-risk telomere length by gravidity group. No difference in odds of having high risk (Table A) or low risk (Table B) telomere length between gravidity groups.
(DOCX)
