# Supplementary material for: Gravidity is not associated with telomere length in a biracial cohort of middle-aged women: The Coronary Artery Risk Development in Young Adults (CARDIA) study
Source: PLoS One. 2017 Oct 19;12(10):e0186495. doi: 10.1371/journal.pone.0186495 (PMC5648190; doi:10.1371/journal.pone.0186495)
Supplement: S1 File — No difference in odds of having high risk (Table A) or low risk (Table B) telomere length between gravidity groups. (DOCX) [file pone.0186495.s001.docx]

**S1 Table A.** No association of gravidity group with high-risk telomere length.

| Gravidity Group | OR (95% CI) | P-value |
| --- | --- | --- |
| 0 Pregnancies | 1.03 (0.61, 1.72) | 0.92 |
| 1 Pregnancy | 0.77 (0.43, 1.38) | 0.38 |
| 2-3 Pregnancies | 1 (ref gp) |  |
| ≥ 4 Pregnancies | 0.73 (0.46, 1.16) | 0.18 |

Odds Ratio (OR) for low-risk (≥ 75^th^ percentile) telomere length. Model includes age and race.

**S1 Table B.** No association of gravidity group with low-risk telomere length.

| Gravidity Group | OR (95% CI) | P-value |
| --- | --- | --- |
| 0 Pregnancies | 0.76 (0.44, 1.44) | 0.35 |
| 1 Pregnancy | 1.37 (0.79, 2.36) | 0.26 |
| 2-3 Pregnancies | 1 (ref gp) |  |
| ≥ 4 Pregnancies | 1.05 (0.68, 1.63) | 0.81 |

Odds Ratio (OR) for high-risk (≤ 25^th^ percentile) telomere length. Model includes age and race.
